# Machine-Learning-Based Survival Prediction in Glioblastoma Using Graph-Theoretical Analysis of Structural Network Alterations

**DOI:** 10.3390/cancers18071161

**Published:** 2026-04-03

**Authors:** Andreas Stadlbauer, Stefan Oberndorfer, Gertraud Heinz, Franz Marhold, Thomas M. Kinfe, Mario Dorostkar, Oliver Schnell, Uwe Meyer-Bäse, Anke Meyer-Bäse

**Affiliations:** 1 Karl Landsteiner University of Health Sciences, 3500 Krems, Austria; stefan.oberndorfer@stpoelten.lknoe.at (S.O.); gertraud.heinz@stpoelten.lknoe.at (G.H.); franz.marhold@stpoelten.lknoe.at (F.M.); mario.dorostkar@kl.ac.at (M.D.); 2 Institute of Medical Radiology, University Clinic of St. Pölten, Karl Landsteiner University of Health Sciences, 3100 St. Pölten, Austria; 3 Department of Neurosurgery, Friedrich-Alexander University (FAU) Erlangen-Nürnberg, 91054 Erlangen, Germany; oliver.schnell@uk-erlangen.de; 4 Department of Neurology, University Clinic of St. Pölten, Karl Landsteiner University of Health Sciences, 3100 St. Pölten, Austria; 5 Department of Neurosurgery, University Clinic of St. Pölten, Karl Landsteiner University of Health Sciences, 3100 St. Pölten, Austria; 6 Mannheim Center for Neuromodulation and Neuroprosthetics (MCNN), Department of Neurosurgery, Mannheim Center for Translational Neuroscience (MCTN), Medical Faculty Mannheim, Heidelberg University, 68167 Mannheim, Germany; thomas.kinfe@umm.de; 7 Institute of Clinical and Molecular Pathology, University Clinic of St. Pölten, Karl Landsteiner University of Health Sciences, 3100 St. Pölten, Austria; 8 Department of Electrical and Computer Engineering, FAMU-FSU College of Engineering, Tallahassee, FL 32310, USA; umb@eng.famu.fsu.edu; 9 Department of Scientific Computing, Dirac Science Library, Florida State University, Tallahassee, FL 32306, USA; ameyerbaese@fsu.edu

**Keywords:** glioblastoma, overall survival, structural connectome, graph-theoretical analysis, artificial intelligence, machine learning, neurooncology

## Abstract

**Background**: Glioblastoma is an extremely aggressive brain tumor that diffusely infiltrates white matter and alters large-scale brain connectivity. Most prognostic models focus on localized tumor features and clinical variables, overlooking broader effects on the brain’s structural connectome. This study addressed this limitation by integrating graph-theoretical analysis of preoperative diffusion tensor imaging (DTI)-derived structural connectomes with machine learning (ML) to improve prediction of overall survival (OS) in newly diagnosed glioblastoma. **Methods**: Preoperative DTI data from 871 glioblastoma patients from the UPenn-GBM and UCSF-PDGM cohorts were processed to construct whole-brain structural connectomes weighted by tract count and quantitative anisotropy (QA). Global and nodal graph-theoretical network metrics were extracted and combined with demographic and clinical information. Ten ML models were trained and validated on 784 patients (90% of the cohort). The three best-performing algorithms were tested on a held-out cohort of 87 patients (10%). **Results**: Random forest, adaptive boosting, and KStar showed the strongest validation performance. In held-out internal testing, random forest models using degree and QA-weighted strength achieved accuracies of 0.862 and 0.874, with AUROCs of 0.929 and 0.909, for predicting OS beyond one year. Strength and clustering coefficient were key predictors, with over two-thirds of significant nodes localized in the temporal lobe, particularly the parahippocampal, and superior, middle, and inferior temporal gyri. **Conclusions**: Graph-theoretical quantification of structural brain network disruption combined with ML allows accurate prediction of OS in glioblastoma. These results support a network-based conceptualization of the disease and indicate that connectome-derived metrics may complement established prognostic frameworks.

## Simple Summary

Glioblastoma is an extremely aggressive brain cancer, yet survival varies widely among affected patients. Existing prognostic approaches mainly examine tumor characteristics and basic patient factors, offering limited insight into why outcomes differ among patients with similar tumors. This study was driven by the hypothesis that glioblastoma disrupts not only the tumor region itself but also the brain’s wider communication networks, which are critical for normal function. The authors sought to quantify preoperative damage to these networks and evaluate its value for improving survival prediction. Using advanced neuroimaging combined with computational analyses, the study demonstrates a strong association between disruption of key brain connections and patient survival. These results support a shift toward viewing the brain as an integrated network and highlight the potential of network-based approaches for prognostic assessment and treatment planning.

## 1. Introduction

Glioblastoma is the most common and aggressive malignant primary brain tumor in adults and remains associated with a dismal prognosis despite multimodal therapy including maximal safe resection, radiotherapy, and temozolomide-based chemotherapy [1]. Median overall survival typically ranges from roughly 12 to 18 months in newly diagnosed disease, with a 5-year survival rate below 5%, underlining its lethality [2,3]. Because clinical outcomes vary substantially between individuals due to patient specific, anatomical, molecular, and treatment related factors, accurate prediction of survival has become a central challenge in neuro-oncology. Accurate survival prediction is clinically important for guiding personalized surgical and adjuvant treatment decisions, providing realistic prognostic information to patients, and potentially identifying individuals who might benefit from intensified or experimental therapies [4]. Traditionally, prognosis relies primarily on demographic and clinical factors (age, gender, extent of resection), imaging surrogates (tumor size and location) [5], and molecular markers such as isocitrate dehydrogenase (IDH) mutation status and O^6^-methylguanine-DNA methyltransferase (MGMT) promoter methylation [6,7]. However, these models often treat the tumor as an isolated mass, without fully accounting for the broader impact of the tumor on brain network organization [8,9].

A characteristic feature of glioblastoma, which is presumably associated with poor prognosis, is the diffuse infiltration of white matter structures. Glioblastoma cells migrate over considerable distances along myelinated fibers, leading not only to local tissue destruction but also to widespread microstructural disruption of critical association, projection, and commissural pathways [10]. The human brain is organized as a complex structural connectome in which discrete cortical and subcortical regions are interconnected through distributed pathways that support essential cognitive and sensorimotor functions. This complex structural network can be conceptualized with fiber tracts forming the edges connecting the distributed cortical and subcortical nodes enabling integrated information processing across spatially distant brain systems [11]. Accordingly, when GBM invades these pathways, it does not merely exert local mass effects; rather, it induces widespread network disruption that may fundamentally alter global connectivity patterns which are closely linked to neurological decline and may also carry prognostic significance [12,13,14].

Diffusion tensor imaging (DTI) based tractography and structural connectivity matrices offer a principled framework to describe white matter organization at the whole-brain level in glioblastoma patients. By parcellating the brain into regions of interest and estimating streamline counts or microstructural weights between each pair of regions, one can construct subject specific structural connectivity matrices that summarize the configuration of the white matter network. Graph-theoretical analysis of these matrices yields a wide range of quantitative network measures that characterize integration, segregation, and node importance in a biologically interpretable manner. Commonly assessed parameters include global efficiency, characteristic path length, assortativity, and small-worldness as measures of large-scale network organization. In addition, nodal and edge wise measures such as nodal degree and strength, betweenness centrality, eigenvector centrality, and clustering coefficient can be derived as markers of each region’s role and integration within the broader connectome. This framework enables the extraction of diverse quantitative metrics that describe network topology at global, regional, and local levels [15,16,17].

Numerous studies in healthy and diseased populations have shown that these metrics provide highly sensitive biomarkers of network integrity, predicting cognitive performance, neurological function, and clinical outcomes across a variety of neurological disorders [18,19,20]. However, their prognostic value for survival in glioblastoma, especially when derived from structural connectivity, remains insufficiently explored and partly controversial. By applying these parameters, the precise, localized, and global degradation of the white matter network due to glioblastoma can be quantitatively assessed, offering a potentially powerful set of novel biomarkers for prognostic evaluation [21].

The comprehensive network characterization afforded by graph-theoretical analysis comes at the cost of generating a multitude of high dimensional parameters per subject that pose significant analytical challenges. A single structural connectome can yield hundreds to thousands of parameters, many of which exhibit nonlinear interactions and higher order dependencies between network features, clinical variables, and molecular markers [22]. Machine learning (ML) approaches offer powerful tools to address this challenge. By leveraging ensemble methods such as random forests, support vector machines, and gradient boosting capable of integrating large numbers of features, ML models can detect latent relationships within high-dimensional data and generate accurate, individualized survival predictions [23,24].

The present study addresses the gap in glioblastoma prognostication by integrating advanced neuroimaging and network analysis with state-of-the-art machine learning. We hypothesize that quantitative measurements of white matter network disruption, as assessed by graph theory parameters derived from preoperative DTI data, provide more mechanistic prognostic information useful for survival prediction of patients with newly diagnosed glioblastoma. The primary aim of this work was to establish a robust machine learning framework utilizing this high-dimensional graph-theoretical network feature set to predict overall survival with high accuracy. Furthermore, we seek to identify the specific network parameters that contribute most significantly to the predictive model, thereby highlighting the fundamental structural vulnerabilities that dictate patient outcome.

## 2. Materials and Methods

### 2.1. Patient Databases

This retrospective study was approved by the Institutional Review Boards of the Lower Austrian Provincial Government and the Friedrich-Alexander University (FAU) Erlangen-Nürnberg. We used two publicly available databases from The Cancer Imaging Archive (TCIA): (i) the University of Pennsylvania glioblastoma (UPenn-GBM) cohort [25] that comprised a total of 630 glioblastoma patients; and (ii) the University of California San Francisco Preoperative Diffuse Glioma MRI (UCSF-PDGM) cohort [26], which included a total of 501 patients with diffuse glioma. The studies conducted in the public datasets have been approved by their respective ethics committee [25,26]. Only patients with a histologically confirmed isocitrate dehydrogenase (IDH) wildtype glioblastoma were included. Patients with incomplete IDH gene status information were excluded in accordance with the 2021 WHO classification criteria [1]. Additionally, patients with missing overall survival (OS) data were excluded from the analysis. The final cohort comprised 871 patients:

- A total of 498 patients (199 females, 299 males, mean age ± standard deviation 63.4 ± 11.8 years, age range 21–89 years) were included from the UPenn-GBM cohort with a median OS of 387 days (OS range 3–2951 days), and
- A total of 373 patients (152 females, 221 males, 61.7 ± 12.0 years, 21–94 years) were included from the UCSF-PDGM cohort with a median OS 361 days (6–2144 days).

For supervised classification with ML, a binary outcome variable was constructed by dichotomizing OS at 365 days. Of the total 871 patients, 784 (90%) were used for model development, including training and validation, while 87 patients (10%) were used as a held-out internal test set to assess generalization performance. Both training/validation and test data were drawn from the same pooled UPenn-GBM and UCSF-PDGM cohorts. The dataset was randomly partitioned using the Randomize filter in the open-source ML software package Weka (Waikato Environment for Knowledge Analysis; version 3.8.6, University of Waikato, Hamilton, New Zealand) [27]. The demographic and clinical details of the patient cohorts are summarized in Table 1.

### 2.2. DTI Data Acquisition and Preprocessing

From each patient included in the study, the DTI data of the pre-treatment clinical MRI scan was used in their publicly available form. Detailed Information on the acquisition parameters of the DTI data can be found in previous publications [25,26]. For the UPenn-GBM data, a DTI diffusion scheme with 30 diffusion sampling directions and a b-value of 1000 s/mm^2^ was used. The in-plane resolution was 1.72 × 1.72 mm^2^ and the slice thickness was 3 mm. For the UCSF-PDGM data the parameters were as follows: a high angular resolution diffusion imaging (HARDI) diffusion scheme with 55 diffusion sampling directions and a b-value of 2000 s/mm^2^ was used. The in-plane resolution was 2.19 × 2.19 mm^2^ and the slice thickness was 2 mm. The two datasets originate from different institutions and were acquired using non-identical imaging protocols and scanner settings, which introduces potential inter-site heterogeneity that may affect downstream connectomic feature estimation and model performance.

**Figure 1:**
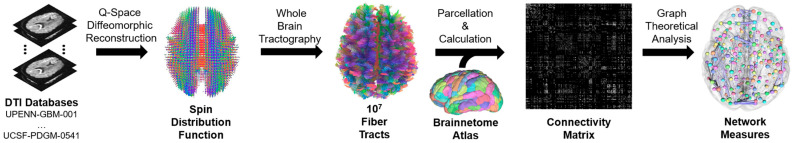
Flowchart of graph-theoretical analysis. The consistent step is the transformation of DTI data into a standardized format using q-space diffeomorphic reconstruction (QSDR). Additionally, the spin distribution function (SDF) is employed to determine the quantity or concentration of water diffusion in different orientations within a particular voxel. For graph-theoretical analysis, deterministic fiber tracking and brain segmentation were used to construct a connectivity matrix. Subsequently, both global and local properties derived from graph theory were calculated. These properties were then used as features for training/validating and testing of machine learning algorithms to predict overall survival of the patients.

The preprocessing of the DTI data was carried out using the open-source software platform DSI Studio (Hou “侯” version; http://dsi-studio.labsolver.org) [28]. Raw DTI data were converted from DICOM to SRC format, followed by quality control in DSI Studio and resampling to an isotropic resolution of 2 mm^3^. Figure 1 illustrates the four-step workflow for DTI data pre- and post-processing.

In the first step, DTI data were spatially normalized by reconstructing them in the common Montreal Neurological Institute (MNI) space. This was performed using the advanced q-space diffeomorphic reconstruction (QSDR) technique in combination with the ICBM152 standard brain template [29,30]. The procedure reassembled each participant’s diffusion data into a universal stereotaxic space and was specifically designed to preserve diffusion spin conservation following nonlinear spatial transformation. Registration quality was assessed by evaluating the R2 values of all normalized files, with a threshold of 0.70 applied to ensure adequate alignment to the template. QSDR enables the calculation of the spin distribution function (SDF) from DTI data, as previously described [30]. The SDF quantifies the amount or density of diffusing water in any direction within a voxel [31,32], thereby characterizing both the directional distribution of water diffusion (reflecting underlying fiber orientations) and the overall density of diffusing spins when integrated across all directions.

In the second step, whole brain tractography was performed using a deterministic fiber tracking algorithm [33] with augmented tracking strategies to enhance reproducibility [34]. The seeding region encompassed the entire brain. Randomly selected thresholds were applied for anisotropy, tracing angle (15° to 90°), and step size (0.5 to 1.5 voxels). Fiber tracts shorter than 30 mm or longer than 300 mm were excluded. In total, 10,000,000 tracts were reconstructed.

In the third step, brain parcellation was performed using the built-in Human Brainnetome Atlas [35]. This atlas comprises 210 cortical and 36 subcortical subregions and was specially developed for fine-grained, connectivity-based structure-to-function mapping that links structural connections to functional patterns. Subsequently, connectivity matrices were computed using both the number of connecting fiber tracts (tract count) and quantitative anisotropy (QA).

Finally, the graph theoretical analysis was performed. Global structural connectivity was evaluated using metrics including density, average clustering coefficient, transitivity, characteristic path length, small-worldness, global efficiency, radius and diameter of graph, assortativity coefficient, and the rich club coefficients k5, k10, k15, and k20. The definitions and biological interpretations of these global structural connectivity measures are provided in Table S1 in the Supplementary Methods Section of the Supplementary Materials. Except for density, both binary (unweighted) and weighted versions of each metric, using tract count or QA as edge weights, were calculated, yielding a total of 27 global network features.

Local structural connectivity was assessed by calculating the unweighted degree of each node in the Brainnetome atlas. In addition, seven weighted local network metrics were computed for all nodes using again tract count or QA as edge weights: strength, clustering coefficient, local efficiency, betweenness centrality, eigenvector centrality, PageRank centrality, and eccentricity. The definitions and biological interpretations of these local structural connectivity measures are provided in Table S2 in the Supplementary Methods Section of the Supplementary Materials.

This yielded eight local network feature vectors, each comprising 246 parameters, corresponding to the two quantitative measures, tract count and QA. Additionally, five demographic and clinical parameters were included for each patient: age, sex, extent of resection, and tumor location (brain hemisphere and lobe). MGMT status was excluded from the analysis due to being missing in over 50% of patients in the UPenn cohort, and Karnofsky Performance Status (KPS) data were not included because they were unavailable for the UCSF-PDGM cohort (Table 1). Methods such as multiple imputation and missing value filling were not used because the missing data in the different cohorts did not follow random patterns and there were not enough covariates to reliably model the mechanisms of the missing data, as this could have led to unpredictable biases. Instead, we prioritized consistently available variables to ensure model integrity across both datasets

### 2.3. Network Feature Selection

Network feature selection was conducted using the Weka software package. For the local network features, a two-step procedure was employed, combining the strengths of both ranking-based and learner-based methods, as described previously [36,37]. In the first step, six different attribute evaluation filters (Correlation, GainRatio, InfoGain, OneR, RefiefF, and SymmetricalUncert) were applied in combination with the Ranker search method. Features that ranked within the top 50 in at least three of the six filter rankings were retained. In the second step, the learner-based Wrapper method was applied with the BestFirst search method, using the specific ML algorithm employed for model development (described in the following section), to further refine the feature list. This process generated an optimized, algorithm-specific feature subset for each classifier. For the global network features, which comprised only 27 features, feature selection was performed solely using the Wrapper method. To address class imbalance, the Synthetic Minority Oversampling Technique (SMOTE) [38] was applied to generate synthetic samples for the minority class.

### 2.4. Model Development and Validation

Model development was also carried out with the Weka software package. Ten commonly used ML algorithms, representing different classifier families, were applied to perform binary classification of overall survival: less than to one year versus greater than or equal to one year. The algorithms included: Naïve Bayes (NB), logistic regression (Log), multilayer perceptron (MP; single hidden layer, number of neurons equal to the sum of features and classes), support vector machine (SVM; with polynomial kernel), k-nearest neighbors (kNN; k = 3), adaptive boosting (Ada; using decision tree “J48” as classifier), decision tree (“J48” in Weka), random forest (RF) [39,40], bootstrap aggregating (Bag; using RandomTree as classifier), and KStar [41]. Brief descriptions of the ML algorithms are provided in the Supplementary Methods Section of the Supplementary Materials.

The unweighted global network measures and the unweighted degree metric were identical when derived from both tract count and QA. Therefore, corresponding models were generated and validated only for tract-count-based network features. For the remaining seven weighted local network metrics, models were trained and validated using both tract-count- and QA-based features. This approach produced a total of 16 distinct graph-theoretical network feature vectors. When combined with the 10 ML algorithms, this resulted in 160 classification models that were trained and validated. Data from 90% of the total patient cohort were allocated for model training and validation. Details of this training/validation cohort are provided in Table 1.

A tenfold cross-validation procedure was employed to validate the models. To strictly prevent information leakage, feature selection and oversampling steps were conducted within each training fold of the cross-validation procedure. Specifically, for each fold, the two-step feature selection and SMOTE rebalancing was applied exclusively to the training data within that fold. The optimized feature subset and trained classifier were then evaluated on the corresponding unseen validation data. This fold-wise process ensured that information from the validation data did not influence feature selection, oversampling, or model training, thereby implementing a fully nested, leakage-safe pipeline [42,43]. Model performance was evaluated using confusion matrix-derived metrics, including accuracy, precision (positive predictive value), sensitivity (recall or true positive rate), specificity (true negative rate), F-score, Youden index, and the area under the receiver operating characteristic curve (AUROC) [44], for predicting OS ≥ 1 year.

### 2.5. Held-Out Internal Model Performance Testing

The three top-performing classifiers were selected for evaluation using held-out internal test data. Data from the remaining 10% of the patient cohort were reserved for this held-out internal testing, with cohort details provided in Table 1. The trained classifier models were saved and subsequently applied to the unseen data from the held-out internal test cohort. Performance was assessed using confusion matrix–derived indicators (i.e., accuracy, precision, sensitivity, specificity, f score, and Youden index), AUROC, and the total number of classification errors. All performance indicators were visualized as heat maps to improve clarity and facilitate comparison across models. Wilson score confidence intervals were calculated for binomial proportions to provide accurate coverage, particularly for small samples or extreme proportions [45].

## 3. Results

### 3.1. Graph-Theoretical Analysis

**Figure 2:**
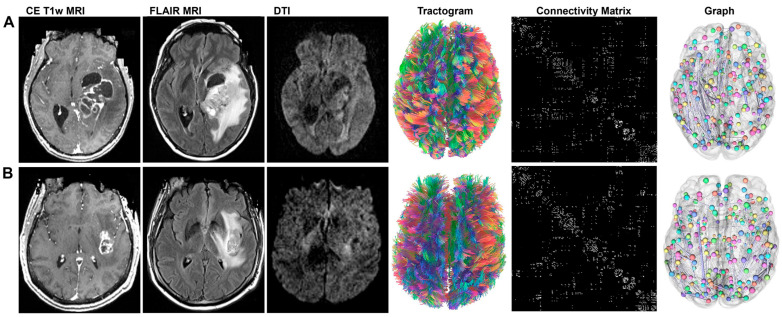
Illustrative cases for graph theoretical analysis. Preoperative MR images including contrast-enhanced (CE) T1-weighted and FLAIR MRI as well as DTI data for (**A**) a 54-year-old female patient with an overall survival of 1882 days (ID: UPENN-GBM-00022) and (**B**) a 64-year-old male patient with an overall survival of 172 days (ID: UPENN-GBM-00043). The DTI data were used to generate whole-brain tractograms from which in turn the connectivity matrices and subsequently the graphs were derived.

Graph-theoretical analysis was successfully completed for all 871 patients selected from the UPenn-GBM and UCSF-PDGM databases. Figure 2 illustrates cases of glioblastoma located in the left temporal lobe, including one patient with overall survival exceeding one year and another with an overall survival under one year. The two tumors were located in a similar region. Despite the tumor in Figure 2A being significantly larger, the patient’s overall survival was much longer (1504 days) compared with 172 days for the patient in Figure 2B. Both patients underwent gross total resection (GTR) with >90% tumor removal. At diagnosis, the patient with the longer survival time was 10 years younger. Differences in the connectivity matrix patterns are observable but subtle and challenging to interpret visually. Additional illustrative cases of glioblastoma located in the right frontal lobe, with OS of 1504 days and 200 days, are presented in Figure S1 in the Supplementary Results Section of the Supplementary Materials. The tumors in these cases were comparable in size and location, but the age difference between the patients (22 vs. 89 years) likely contributed to the survival disparity.

### 3.2. Training, Validation and Selection of Models

The network feature data, together with demographic and clinical data from 784 patients (90% of the total cohort), were used for model training. Figures S2 and S3 in the Supplementary Results Section of the Supplementary Materials provide heatmap overviews of the performance indicators for all 10 ML algorithms applied to graph-theoretical network weighted metrics by the two quantitative measures, tract count and QA. Among the models, adaptive boosting, random forest, and KStar consistently demonstrated superior performance, with validation metrics generally exceeding 0.9 across all network features. Consequently, these three models were selected for held-out internal testing. For comparative analysis, the three top-performing models were also trained and validated using demographic and clinical variables alone. Under these conditions, all indicators of predictive performance were markedly reduced (Table S3 in the Supplementary Results Section of the Supplementary Materials).

### 3.3. Held-Out Internal Testing of the Selected Models

**Figure 3:**
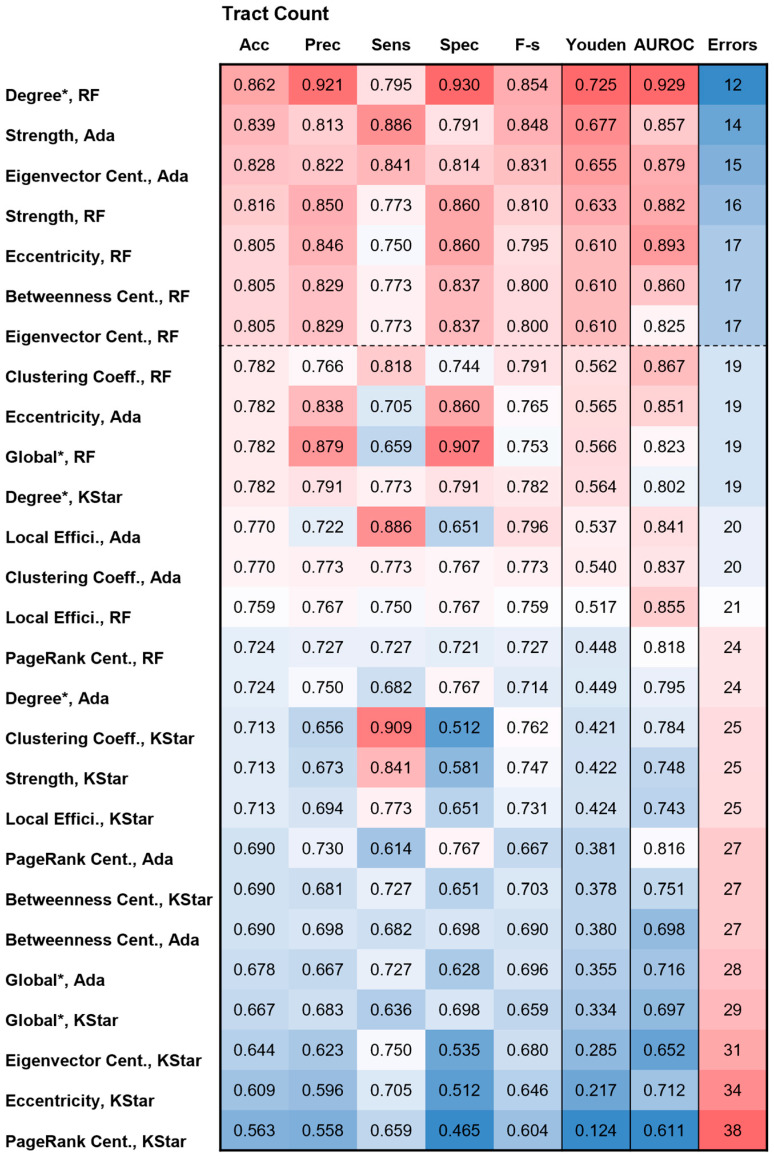
Heatmap of the predictive performance indicators for the held-out internal testing of the three selected models: random forest (RF), adaptive boosting (Ada), and KStar for all graph measures weighted with the parameter tract count. The combinations of graph measure and ML algorithm are sorted from top to bottom according to decreasing Youden index and increasing number of classification errors. The color codes for accuracy (Acc), precision (Prec), sensitivity (Sens), specificity (Spec), and F-score (F-s.) are identical, ranging from a minimum of 0.46 (blue) to a maximum of 0.93 (red). Separate color codes exist for Youden index, AUROC, and classification errors due to their different value ranges. * The global network measure and local network measure degree is an unweighted parameter and therefore identical for both tract count and QA, resulting in identical performance indicators during held-out internal testing.

**Figure 4:**
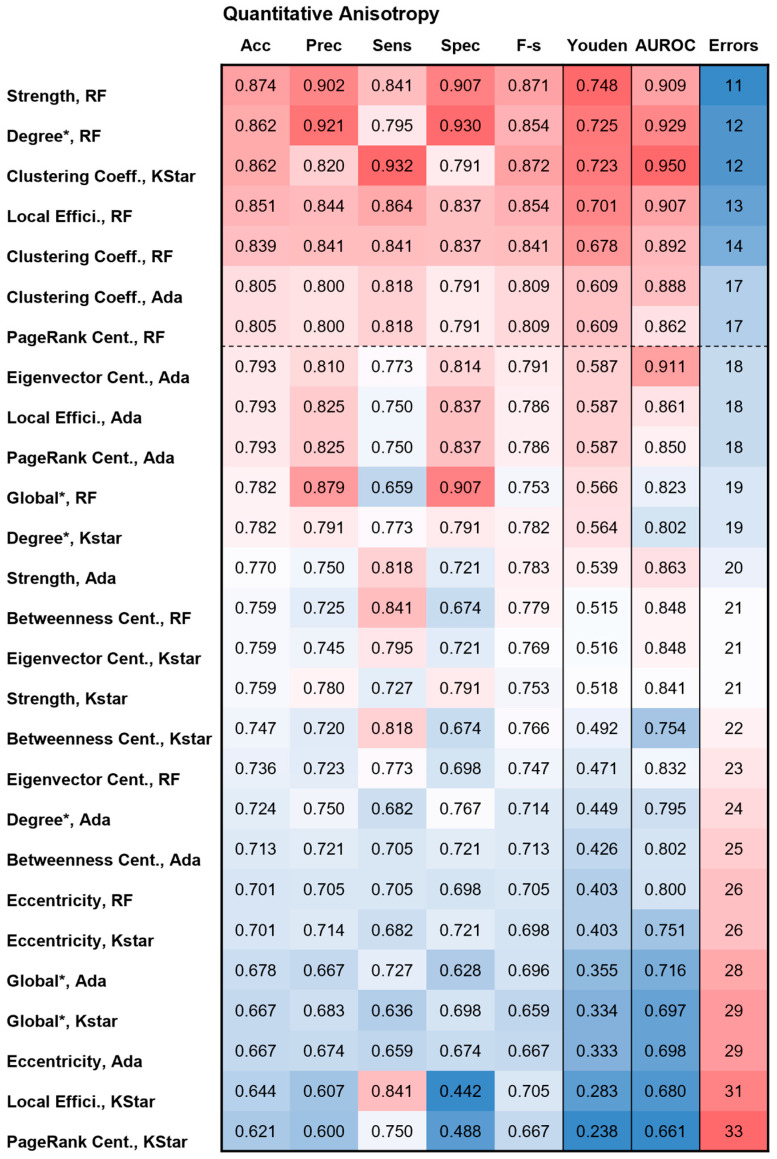
Heatmap of the predictive performance indicators for the held-out internal testing of the three selected models: random forest (RF), adaptive boosting (Ada), and KStar for all graph measures weighted with the parameter quantitative anisotropy (QA). The combinations of graph measure and ML algorithm are sorted from top to bottom according to decreasing Youden index and increasing number of classification errors. The color codes for accuracy (Acc), precision (Prec), sensitivity (Sens), specificity (Spec), and F-score (F-s.) are identical, ranging from a minimum of 0.44 (blue) to a maximum of 0.93 (red). Separate color codes exist for Youden index, AUROC, and classification errors due to their different value ranges. * The global network measure and local network measure degree is an unweighted parameter and therefore identical for both tract count and QA, resulting in identical performance indicators during held-out internal testing.

The network feature data, together with demographic and clinical variables from 87 patients (10% of the total cohort), were used for held-out internal testing of the top-performing models identified during training and validation: adaptive boosting, random forest, and KStar. Figure 3 and Figure 4 present heatmaps of the performance indicators for these three models applied to all graph-theoretical network measures weighted by tract count and QA, respectively. The combinations of network measure and algorithm are listed on the far left and sorted in descending order according to the Youden index and classification error.

The local network measure degree is an unweighted parameter and therefore identical for both tract count and QA, resulting in identical performance indicators during held-out internal testing. When combined with the random forest algorithm, degree achieved the best performance for the tract count parameter, with an accuracy of 0.862, precision of 0.921, Youden index of 0.725, AUROC of 0.929, and 12 classification errors. For the combination of the QA-weighted local network measure strength and the random forest model, performance was slightly improved in terms of accuracy (0.874), Youden index (0.748), and classification errors (11) but slightly lower in precision (0.902) and AUROC (0.909). Additional combinations of network measures and models achieving a Youden index above 0.6 and fewer than 17 classification errors (<20%) were observed for both tract-count- and QA-weighted features. Tables S4 and S5 (Supplementary Results Section of the Supplementary Materials) demonstrate that the confidence intervals for some metrics overlap between different models, indicating that apparent small differences in point estimates may not be statistically meaningful.

For comparison, the three top-performing models trained demographic and clinical variables alone were also tested using only the demographic and clinical data, resulting again in markedly lower predictive performance across all evaluated metrics (Table S6 in the Supplementary Results Section of the Supplementary Materials). However, it should be explicitly noted that key prognostic variables such as MGMT status and KPS were not included due to substantial and non-uniform missingness across the combined cohorts. Consequently, comparisons between clinical and connectome-based models must be interpreted with appropriate caution and are inherently limited.

**Figure 5:**
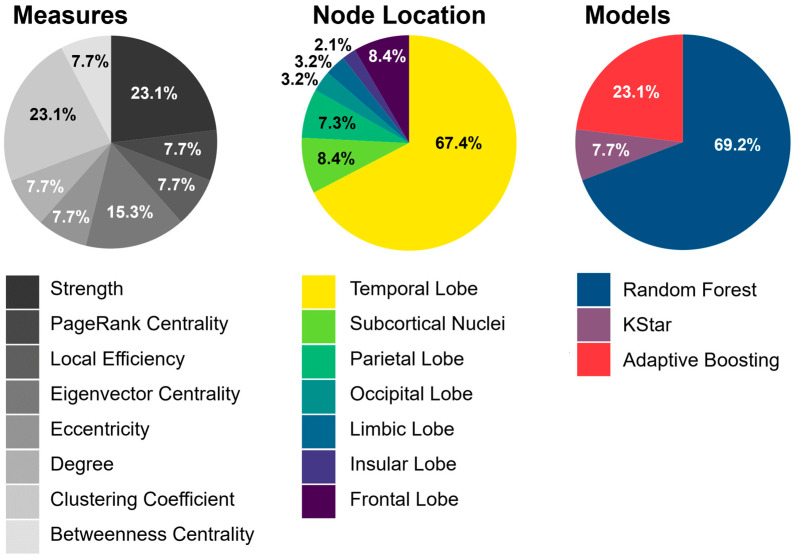
Overview of the best-performing graph-theoretical network measures and their node locations as well as ML models for predicting overall survival for more than one year.

Figure 5 presents the 13 top-performing combinations of network metrics and ML models. Among the graph-theoretical measures, strength and clustering coefficient showed most often (23.1% each) a strong predictive performance, followed by eigenvector centrality (15.3%). More than two-thirds (67.4%) of the nodes identified as particularly informative for prediction were located in the temporal lobe, with the parahippocampal gyrus most frequently represented, followed by the superior, middle, and inferior temporal gyri. The most commonly implicated subcortical structure was the thalamus, while within the frontal lobe the precentral gyrus was predominant. With respect to machine learning methods, random forest clearly outperformed other approaches, accounting for nearly 70% of the top-performing models.

## 4. Discussion

This study shows that preoperative structural connectome integrity, assessed through graph-theoretical analysis of DTI-derived networks, is a promising predictor of overall survival in patients with newly diagnosed glioblastoma. Incorporating these high-dimensional network features into machine learning models yielded high predictive performance and revealed distinct topological signatures of white matter disruption associated with patient survival. These results corroborate with our central hypothesis that the diffuse infiltration typical of glioblastoma causes extensive network disturbances with significant prognostic relevance, may provide information beyond conventional factors such as tumor volume or patient age. Therefore, glioblastoma should be conceptualized as a disease of distributed network failure, in which survival is closely linked to the integrity of large-scale white matter connectivity.

Our results are particularly noteworthy in three respects: graph-theoretical network measures, node localization, and machine learning models. A central finding of our study was the robust and consistent predictive value of local graph measures, particularly nodal degree and strength, across both weighting schemes and multiple ML algorithms. These measures capture the quantity and intensity of a node’s connections, indicating its topological relevance and role in facilitating information flow. Furthermore, QA-weighted clustering coefficient and tract-count-weighted eigenvector centrality also demonstrated promising prognostic relevance when incorporated into the ML models. Clustering coefficient indexes local segregation and connection redundancy, whereas eigenvector centrality characterizes a node’s influence within the global network by emphasizing connections to other highly connected nodes. This observation is consistent with prior reports indicating that glioma infiltration reduces local network efficiency and modular integrity, thereby limiting compensatory reorganization and contributing to clinical deterioration [46,47,48,49]. The prominence of these measures within the predictive models indicates that survival in glioblastoma is strongly dependent on the preservation of highly connected network hubs and locally clustered subnetworks. Tumor-induced infiltration or disconnection of these central nodes may precipitate large-scale network failure, undermining compensatory mechanisms and contributing to rapid functional deterioration and mortality. Importantly, global graph metrics did not rank among the most promising predictors. This likely reflects the pronounced heterogeneity of glioblastoma-related network disruption, whereby regional vulnerability conveys greater prognostic information than coarse summaries of global network topology. The slightly superior performance of QA-weighted graph measures may reflect the greater sensitivity of QA to intra-voxel anisotropy and fiber density, thereby mitigating the limitations of streamline count–based measures that are susceptible to tractography biases [36,50]. QA has been proposed to more accurately reflect microstructural integrity and fiber coherence than streamline count alone, which is known to be susceptible to tractography-related biases [51]. This observation motivates future work employing refined diffusion-based weighting schemes, such as q-space imaging (QSI), to more precisely characterize microstructural integrity and connectivity strength in tumor-disrupted networks.

The anatomical distribution of prognostically relevant nodes further reinforces the biological plausibility of our results. More than two-thirds of the most informative nodes were located in the temporal lobe, with particular prominence in the parahippocampal gyrus and the superior, middle, and inferior temporal gyri. These regions serve as major convergence hubs for long-range association fibers, including the inferior longitudinal fasciculus, inferior fronto-occipital fasciculus, and uncinate fasciculus [52]. Disruption of these pathways has previously been associated with cognitive impairment, increased seizure burden, and reduced quality of life in glioma patients [53], factors that are themselves linked to poorer survival outcomes [54]. Luckett et al. [55] employed demographic variables, cortical thickness measures, and resting-state functional network connectivity to train a deep neural network for predicting survival in glioblastoma patients. Their analysis identified cortical thickness in the parahippocampal gyrus, superior temporal sulcus, and middle temporal regions as among the most promising predictors of survival.

Noteworthy is also the prominent contribution of subcortical structures, particularly the thalamus, to survival prediction. As a central relay hub linking cortical and subcortical systems, thalamic disconnection can exert widespread effects on consciousness, cognition, and motor function [56]. Previous structural and functional connectivity studies have linked thalamocortical disintegration to rapid neurological decline and poor prognosis in high-grade gliomas [57,58]. Our findings extend these observations by showing that quantitative measures of thalamic network embedding provide prognostic information at the population level.

In the frontal lobe, the precentral gyrus also emerged as a prognostically relevant node. As the cortical anchor of the primary motor network, its structural disconnection is likely to manifest as early motor deficits, reduced KPS, and diminished tolerance for aggressive treatment, all of which are well-established predictors of shorter survival [59]. The convergence of these anatomical observations with known clinical trajectories supports the view that network metrics index meaningful pathophysiological processes rather than spurious associations.

Among the ML approaches evaluated, random forest models consistently achieved the highest predictive performance, representing nearly 70% of the top-performing network-feature-model combinations. This aligns with previous neuro-oncological applications of ensemble learning, where random forests have demonstrated robustness to multicollinearity, nonlinear interactions, and noisy features [60,61]. The promising performance observed here further supports the suitability of tree-based ensemble methods for modeling high-dimensional connectomic data.

The illustrative cases described further underscore the added value of network-based analysis. Patients with tumors of comparable size and location but markedly different survival displayed subtle yet significant differences in connectivity patterns that are not readily apparent on visual inspection. This emphasizes a key strength of graph-theoretical and ML approaches: their capacity to identify distributed, multivariate signatures of disease burden that elude conventional radiological assessment.

Traditional survival prediction models for glioblastoma that rely mainly on clinical variables (such as age and extent of resection) and core molecular markers (including IDH mutation status and MGMT promoter methylation) generally achieve only moderate discrimination, with AUROC values around 0.71–0.75 [9]. More recent radiomics and ML approaches have enhanced performance by leveraging high-dimensional feature sets derived from structural MRI. Duman et al. [62] developed integrated clinical and MRI-based radiomic models for predicting OS in glioblastoma patients utilizing the publicly available BraTs database. Their best-performing prognostic model, which incorporated regularized Cox regression with gross tumor volume, age, and FLAIR MRI features, achieved an AUROC of 0.81 in the validation cohort. Karabacak et al. [63], similarly to our study, utilized the PENN-GBM and UCSF-PDGM databases to develop and evaluate a gradient boosting model based on radiomic features derived from anatomical MRI sequences (native T1w, ceT1w, T2w, FLAIR). For predicting 6-month overall survival, their model achieved an AUROC of 0.79. Marasi et al. [64] combined anatomical MRI data from the BraTS database with patient age as a clinical variable to train a multi-layer perceptron. Their model achieved the highest performance for predicting 400-day survival, with an AUROC of 0.74 and an accuracy of 0.71. All three studies relied on MRI data from publicly available databases with large patient cohorts, similar to our approach, and employed machine learning algorithms comparable to those used in our work.

Our findings complement and extend this literature by shifting the analytical focus from the tumor itself to its effects on the surrounding brain networks. This conceptual transition from lesion-centric to network-based prognostication mirrors broader trends in neurological research, as seen in connectomic biomarkers for stroke, epilepsy, and neurodegenerative disorders [17,65]. In glioblastoma specifically, recent structural and functional connectomics studies have linked connectome disruption, hub connectivity alterations, and topological reorganization to impaired cognitive outcomes and reduced survival [12,66].

Gopalakrishnan et al. [67] developed a 4D-tumor-connectomics framework to characterize glioblastoma topology across multiparametric MRIs obtained preoperatively, postoperatively, at first post-radiation therapy follow-up, and at progression. They extracted 88 connectomic features from the longitudinal MRI sequences and combined these with 20 clinical and treatment variables, including age, KPS, MGMT methylation status, and presence of radiation necrosis. A support vector machine model was employed to distinguish overall survival differences. Their model achieved a Youden index of 0.77 and an AUROC of 0.98 for predicting survival under 1 year. Among graph measures, degree, clustering coefficient, and betweenness centrality were most predictive. The approach, methodology, and principal results of this study closely parallel our own. Their results slightly surpassed ours, likely because they relied on leave-one-out cross-validation rather than held-out internal testing. Nonetheless, their findings reinforce the value of incorporating longitudinal MRI data and a broader set of clinical covariates to enhance survival prediction.

From a clinical perspective, accurate preoperative survival prediction using network-based biomarkers offers several important implications. These metrics could enable individualized risk stratification at diagnosis and facilitate patient selection for intensified or experimental therapies. Moreover, identifying specific network vulnerabilities may inform surgical planning to preserve critical connections, consistent with emerging principles of connectome-guided neurosurgery.

Although this study benefits from a large, cross-institutional dataset, several methodological limitations warrant consideration. First, DTI-based tractography, while widely available in clinical practice, is limited in its ability to resolve crossing fibers and may underestimate connectivity in complex white matter pathways. Advanced diffusion data acquisition and processing techniques, such as Diffusion Spectrum Imaging and Generalized Q-Sampling Imaging, could improve the accuracy and reliability of network reconstruction [31]. Because the diffusion tensor model cannot fully resolve complex crossing fiber configurations, our connectomes likely underestimate connectivity in such regions. Future studies using advanced multi shell diffusion and higher order models may further refine network characterization, improve prognostic accuracy, and further enhance survival prediction. Second, our network models did not incorporate molecular markers, postoperative complications, or clinical data obtained after radiochemotherapy. Future studies should examine longitudinal network changes during and following treatment, as these may serve as dynamic biomarkers of therapy response and disease progression. Third, the choice of parcellation scheme for defining network nodes can substantially affect the resulting graph metrics. We employed the Brainnetome atlas, which, although relatively new and less widely adopted than traditional atlases, offers a modern, connectivity-based framework that integrates multimodal data and provides a more functionally informed representation of brain architecture. However, applying a template derived from healthy brains to patients with space occupying lesions introduces potential misregistration and label displacement, especially near the tumor and edematous regions. To limit these effects, we performed normalization using a dedicated q space diffeomorphic reconstruction framework with explicit quality control criteria, and our network level analyses aggregate information across many nodes, which performs nonlinear spatial transformation while preserving diffusion information. This step ensures that the atlas-based parcellation is applied within a standardized coordinate framework. Developing a standardized atlas specifically adapted for tumor-bearing brains could help reduce variability across studies. Fourth, survival was modeled as a binary classification task (OS < 1 year vs. ≥ 1 year), which holds clear clinical relevance. Survival modeling approaches, such as Cox proportional hazards models or survival-based machine learning methods, could offer deeper insight. Future work should therefore prioritize time-to-event modeling frameworks as a key methodological extension, enabling more refined and clinically informative prognostic assessment. Fifth, missing MGMT status in over half of UPenn-GBM cohort and complete absence of KPS data in UCSF-PDGM cohort limit direct comparability with single-center studies using comprehensive molecular and functional covariates in established prognostic frameworks. Sixth, a further limitation is the heterogeneity between the two source cohorts, which may reflect differences in scanner platforms, acquisition protocols, preprocessing conditions, and clinical data availability. Although all data were processed using a uniform pipeline and the cohort was randomly partitioned for model development and testing, residual site-related effects cannot be excluded. Therefore, the reported performance should be interpreted as internal validation across pooled multicenter data rather than definitive evidence of generalizability, and external validation in independent cohorts is required. Finally, the high dimensionality of connectomic data poses an overfitting risk. We mitigated this through rigorous feature selection, ensemble algorithms, and held-out internal testing. However, prospective external validation remains essential prior to clinical implementation. Future work could incorporate robust representation-learning and robustness-oriented preprocessing to derive more stable, noise-resilient graph-theoretical embeddings that generalize better across scanners and acquisition protocols.

## 5. Conclusions

In conclusion, our study demonstrates that graph-theoretical analysis of structural connectomes, integrated with machine learning, reveals glioblastoma-induced white matter network disruptions as promising prognostic markers of overall survival. By redirecting focus from the tumor mass to the broader brain network, we identified critical structural vulnerabilities especially in the temporal lobe, the frontal lobe and thalamus that significantly influence patient outcomes. These findings support a network-based conceptualization of glioblastoma and highlight specific anatomical and topological targets for future prognostication, treatment planning, and mechanistic investigation.

## Figures and Tables

**Table 1:** Demographic and clinical details of the patient cohorts.

	UPenn-GBM	UCSF-PDGM	Training/ Validation	Held-Out Internal Testing
Total number of patients	498	373	784	87
Age (mean ± sd)	63.4 ± 11.8 yrs	61.7 ± 12.0 yrs	62.9 ± 11.8 yrs	60.6 ± 12.6 yrs
Age Range	21–89 yrs	21–94 yrs	21–94 yrs	33–88 yrs
Gender: female	199 (40.0%)	152 (40.8%)	320 (40.8%)	31 (35.6%)
Gender: male	299 (60.0%)	221 (59.2%)	464 (59.2%)	56 (64.4%)
Overall survival median	387 d	361 d	376 d	365 d
Overall survival range	3–2951 d	6–2144 d	3–2951 d	17–2021 d
Extent of Resection: GTR	288 (57.8%)	218 (58.4%)	449 (57.3%)	57 (65.5%)
Extent of Resection: STR	172 (34.6%)	155 (41.6%)	300 (38.3%)	27 (31.0%)
Extent of Resection: NA	38 (7.6%)	-	35 (4.4%)	3 (3.5%)
MGMT unmethylated	135 (27.1%)	105 (28.2%)	208 (26.5%)	32 (36.8%)
MGMT methylated	112 (22.5%)	256 (68.6%)	325 (41.5%)	43 (49.4%)
NA	251 (50.4%)	12 (3.2%)	251 (32.0%)	12 (13.8%)
KPS ≥ 80	60 (12.1%)	-	55 (7.0%)	5 (5.8%)
KPS < 80	13 (2.6%)	-	12 (1.5%)	1 (1.1%)
NA	425 (85.3%)	373 (100%)	717 (91.5%)	81 (93.1%)
patients with OS ≥ 1a	270 (54.2%)	183 (49.1%)	409 (52.2%)	44 (50.6%)
patients with OS < 1a	228 (45.8%)	190 (50.9%)	375 (47.8%)	43 (49.4%)

KPS—Karnofsky Performance Score; GTR—gross total resection; STR—subtotal resection (<90% resection); NA—not available; MGMT—O^6^-methylguanine-DNA methyltransferase; yrs—years; d—days.

## Data Availability

These data were derived from the following resources available in the public domain: [The University of Pennsylvania glioblastoma (UPenn-GBM) cohort, https://www.cancerimagingarchive.net/collection/upenn-gbm/ (accessed on 3 March 2026) and The University of California San Francisco Preoperative Diffuse Glioma MRI (UCSF-PDGM) cohort, https://www.cancerimagingarchive.net/collection/ucsf-pdgm/ (accessed on 3 March 2026).

## Supplementary Materials

The following supporting information can be downloaded at: https://www.mdpi.com/article/10.3390/cancers18071161/s1, Table S1: Global Structural Connectivity Measures; Table S2: Local Structural Connectivity Measures; Figure S1: Illustrative cases for graph-theoretical analysis; Figure S2: Heatmap of the predictive performance indicators for training and validation Part 1; Figure S3: Heatmap of the predictive performance indicators for training and validation Part 2; Table S3: Indicators of predictive performance for the top-performing models trained and validated using only demographic and clinical data; Table S4: Predictive performance indicators and 95% confidence intervals for the held-out internal testing using graph measures weighted with the parameter tract count; Table S5: Predictive performance indicators and 95% confidence intervals for the held-out internal testing using graph measures weighted with the parameter QA; Table S6: Indicators of predictive performance for the top-performing models tested using only demographic and clinical data. Reference [68] is cited in the supplementary materials.

## Abbreviations

The following abbreviations are used in this manuscript:

ML	machine learning
DTI	diffusion tensor imaging
OS	overall survival
QA	quantitative anisotropy
AUROC	area under the receiver operating characteristic curve
IDH	isocitrate dehydrogenase
MGMT	O^6^-methylguanine-DNA methyltransferase
TCIA	The Cancer Imaging Archive
UPenn-GBM	University of Pennsylvania glioblastoma
UCSF-PDGM	University of California San Francisco Preoperative Diffuse Glioma MRI
HARDI	high angular resolution diffusion imaging
DICOM	Digital Imaging and Communications in Medicine
QSDR	Q-space diffeomorphic reconstruction
SDF	spin distribution function
MNI	Montreal Neurological Institute
KPS	Karnofsky Performance Status
SMOTE	Synthetic Minority Oversampling Technique
NB	naïve Bayes
Log	logistic regression
MP	multilayer perceptron
SVM	support vector machine
kNN	k-nearest neighbors
Ada	adaptive boosting
RF	random forest
Bag	bootstrap aggregating
GTR	gross total resection
STR	subtotal resection
ceT1w	contrast-enhanced T1-weighted
FLAIR	Fluid-Attenuated Inversion Recovery
Acc	accuracy
Prec	precision
Sens	sensitivity
Spec	specificity
F-s	F-score
NA	not available
