# Supplementary material for: Machine-Learning-Based Survival Prediction in Glioblastoma Using Graph-Theoretical Analysis of Structural Network Alterations
Source: Cancers (Basel). 2026 Apr 3;18(7):1161. doi: 10.3390/cancers18071161 (PMC13072249; doi:10.3390/cancers18071161)
Supplement: Supplementary file 1 [file cancers-18-01161-s001.zip › cancers-4209093-supplementary.pdf]

Machine-Learning-Based Survival Prediction in Glioblastoma Using Graph-Theoretical Analysis of Structural Network Alterations

Supplementary Materials

Supplementary Methods

Table S1. Global Structural Connectivity Measures.

Measure	Definition	Biological Meaning
**Density**	Fraction of existing edges relative to all possible edges in the network	Indicates how globally connected the brain network is. A higher density indicates more reconstructed fiber tracts between regions
**Average Clustering Coefficient**	Average probability that two neighbors of a node are also connected with each other.	Captures the tendency for local neighborhoods of brain regions to form tightly interconnected clusters, interpreted as structural support for local specialization.
**Transitivity**	Ratio of closed triangles to total connected node triplets in the network	Reflects the overall level of clustering at the network level. Interpreted as the brain’s capacity for cohesive local information processing.
**Characteristic Path Length**	The average shortest path length between all pairs of nodes in the network.	Measures the global efficiency of information transfer. Shorter path lengths facilitate faster and more efficient communication across distant brain regions.
**Small-Worldness**	A measure comparing the network's clustering coefficient and path length to those of a comparable random network	Indicates an optimal balance between local specialization and global integration, which is characteristic of the healthy, efficient brain network architecture.
**Global Efficiency**	The average inverse of the shortest path lengths across all node pairs.	A direct measure of how efficiently information is exchanged across the whole brain network
**Radius of Graph**	The minimum of the greatest shortest-path distance from a node to any other node.	Identifies the most centrally embedded region(s) in the network. A smaller radius implies at least one region is acting as a central hub of integration
**Diameter of Graph**	The longest shortest path in the entire network.	Captures the size of the network in topological terms. Smaller diameters reflect compact communication topology.
**Assortativity Coefficient**	A Measure of the tendency of nodes to connect to other nodes with similar degree (or strength).	Reflects the resilience and stability of the network. High assortativity suggests a robust core of interconnected hubs, which, if targeted, could disrupt global communication significantly.
**Rich Club Coefficients (*k5, k10, k15, k20*)**	Quantifies the density of connections among nodes whose degree exceeds a threshold *k.*	Measures the presence and strength of a high-degree “core” of regions (rich club) that form an integrative backbone of the brain.

Taken from Sporns and Kötter [1].

Table S2. Local Structural Connectivity Measures.

Measure	Definition	Biological Meaning
**Degree**	The number of direct connections (edges) a node has to other nodes in the network.	Represents how many direct anatomical neighbors a region has in the connectome. Nodes with high degree are structural hubs with many direct white-matter connections
**Strength**	The sum of the weights (tract count, QA) of all edges connected to a node, i.e. weighted degree.	Reflects how strongly a region is structurally connected to the rest of the brain. High-strength nodes support substantial information throughput and may act as weighted hubs.
**Clustering Coefficient**	The fraction of a node's neighbors that are also connected to each other.	Reflects local specialization and the presence of tightly interconnected subcircuits. High clustering suggests involvement in specialized or modular processing.
**Local Efficiency**	Efficiency of communication between a node's neighbors when that node is removed.	Indicates the resilience of local information processing and redundancy in the immediate neighborhood. High values suggest robust local circuitry even under node failure.
**Betweenness Centrality**	The proportion of all network shortest paths that pass through a given node.	Identifies nodes acting as bridges or bottlenecks connecting different subnetworks. High betweenness regions facilitate integrative communication and may be critical for global information routing.
**Eigenvector Centrality**	Measure of influence based on the principle that connections to highly central nodes contribute more to a node's centrality score.	Highlights nodes embedded within influential, high-connectivity cores. Such regions are often critical for global integration and coordination.
**PageRank Centrality**	Variant of eigenvector-like centrality that distributes importance via incoming connections with damping	Emphasizes regions that receive many or strong connections from already important areas. Highlighting structurally influential nodes that may disproportionately shape large-scale communication
**Eccentricity**	The maximum shortest-path distance from a node to any other node in the network.	Reflects the node's topological position relative to the rest of the brain network. Low eccentricity indicates a centrally located region.

Taken from Sporns and Kötter [1].

Machine Learning Algorithms

Naive Bayes. Bayes decision theory represents a fundamental statistical approach in pattern classification. This classifier is based on the assumption that the decision problem is formulated in probabilistic terms, and that all relevant probability values are given. Simply formulated, the probability that a pattern belongs to a given class is determined.

Logistic regression. Logistic regression represents a statistical model employing the logistic function for modeling a binary dependent variable. It unveils the relationship between the dependent variable and one or more independent variables based on estimating probabilities employing the logistic regression equation.

Multilayer perceptron (MP). The architecture of the MP is completely defined by an input layer, one or more hidden layers, and an output layer. The input data is processed by the MP in a forward direction, passing through each single layer. The training of the network is accomplished based on a supervised learning technique (backpropagation) that requires given input-output data pairs.

Support vector machine (SVM). SVMs are supervised learning models for classification and regression analysis. Applied to a two-class problem, they map the training data such as to maximize the width of the gap between the two classes. They can be performed both linear and nonlinear classification. Non-linear classification is achieved via the kernel trick, which implicitly maps inputs into high-dimensional feature spaces. Kernels can be polynomial, sigmoid, or Gaussian.

k-nearest neighbor (kNN). kNN is a supervised classifier. This algorithm stores all available patterns and classifies new patterns based on a similarity measure (e.g., distance functions) and majority class of the patterns in the specified number of neighbors.

Adaptive boosting (Ada). Ada is an ensemble learning method and can be used in conjunction with many other types of learning algorithms to improve performance. The output of the other learning algorithms ('weak learners') is combined into a weighted sum that represents the final output of the boosted classifier. Ada is adaptive in the sense that subsequent weak learners are tweaked in favor of those instances misclassified by previous classifiers. Ada typically combines weak learners (such as decision stumps) but can also effectively combine strong learners (such as deep decision trees).

Decision tree. Decision trees represent a nonlinear multistage classifiers in which classes are rejected over a sequence of decisions until a finally accepted class is reached. This means that the feature space is split sequentially in specific regions which correspond to the classes. Each feature vector traverses an existing tree based on a sequence of decisions

and follows a path of nodes until it reaches the region where it belongs. In other words, the correct class corresponding to a feature vector is determined by searching a tree–based decision system. This classification scheme is extremely beneficial when a large number of classes is given. The most popular decision trees are binary decision trees.

Random forest. The random forest is an ensemble method composed of many smaller models. The classification and prediction is achieved by combining the outputs of these smaller models which are usually classification and regression trees (CART). CART operates based on a repeated partitioning of the input data in order to estimate the conditional distribution of a response (output class) for a given set of feature variables. The algorithm implements a binary decision tree where every single feature of the input is considered as a candidate for the split. Binary decision trees are nonlinear multistage classifiers that operate by searching a tree-based decision system. The trees are combined to a forest based on bagging. To avoid overfitting, each model is fitted only to a sample of the same size as the original input data but selected with replacement. This sample technique is known as the bootstrap sample.

Bootstrap aggregating (Bag). Bag is an ensemble machine learning algorithm. It is designed to improve stability and accuracy of machine learning algorithms for both classification and regression. Bag is commonly used to reduce variance within a noisy dataset and helps to avoid overfitting. Bag is composed of two parts: bootstrapping and aggregation. Bootstrapping is a sampling method, where a sample is randomly chosen out of a set, using the replacement method. The learning algorithm is then run on the samples selected. The outputs from all the separate models are aggregated into a single prediction as part of the final model. Bagging is usually applied to decision tree methods but can also be used with any type of machine learning method.

KStar. KStar is an information-theoretic, instance-based (lazy) classification method that replaces geometric (Euclidean or Hamming) distance with entropy-based similarity, allowing it to model complex attribute relationships without explicit feature scaling or model training. KStar stores the training instances and classifies a new instance by examining how similar it is to all stored instances in the training set, similar to kNN, but it measures similarity using the probability of transforming one instance into another. The central idea is an entropic distance measure in which two instances are considered similar when one can be transformed into the other through a sequence of simple operations with high probability, corresponding to low entropy.

Supplementary Results

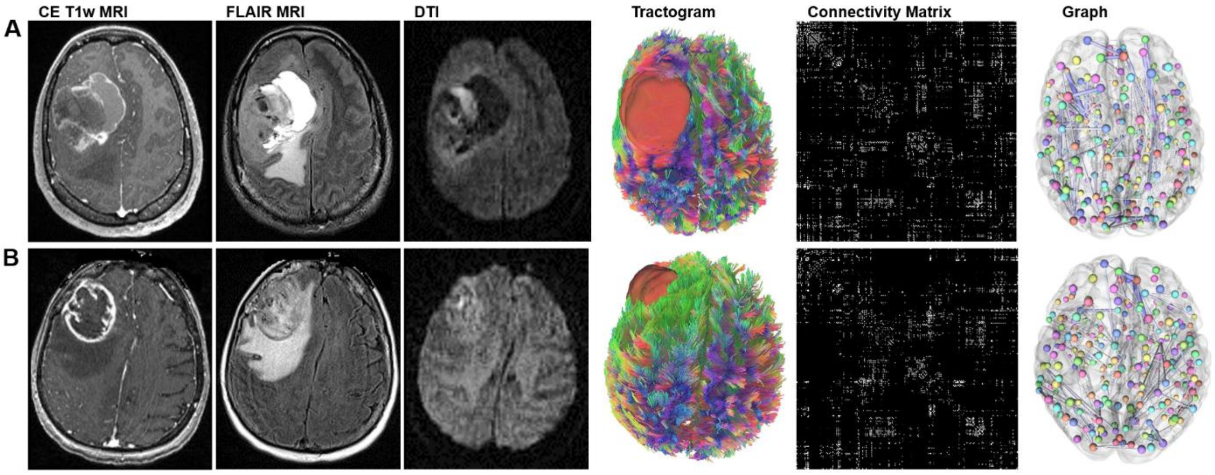

Figure S1. Illustrative cases for graph-theoretical analysis. Preoperative MR images including contrast-enhanced (CE) T1-weighted and FLAIR MRI as well as DTI data for **A**) a 22-year-old female patient with an overall survival of 1504 days (ID: UPENN-GBM-00029) and **B**) an 89-year-old female patient with an overall survival of 200 days (ID: UPENN-GBM-00185). The DTI data were used to generate whole-brain tractograms (tumors depicted as red volumes), from which in turn the connectivity matrices and subsequently the graphs were derived.

A

Global

	NB	Log	MP	SVM	kNN	Ada	J48	RF	Bag	KStar
Accuracy	0.731	0.706	0.840	0.722	0.877	0.910	0.859	0.914	0.897	0.910
Precision	0.725	0.704	0.853	0.718	0.887	0.908	0.859	0.931	0.901	0.925
Sensitivity	0.746	0.712	0.821	0.731	0.863	0.913	0.859	0.893	0.891	0.893
Specificity	0.717	0.700	0.858	0.713	0.890	0.907	0.859	0.934	0.902	0.927
F score	0.735	0.708	0.837	0.724	0.875	0.910	0.859	0.912	0.896	0.909
Youden	0.463	0.412	0.679	0.444	0.753	0.820	0.718	0.827	0.793	0.820
AUROC	0.786	0.761	0.883	0.722	0.928	0.966	0.872	0.972	0.954	0.972

Degree

	NB	Log	MP	SVM	kNN	Ada	J48	RF	Bag	KStar
Accuracy	0.695	0.731	0.780	0.749	0.889	0.907	0.877	0.899	0.888	0.901
Precision	0.652	0.719	0.793	0.739	0.899	0.915	0.876	0.904	0.892	0.893
Sensitivity	0.840	0.758	0.757	0.769	0.875	0.897	0.878	0.893	0.883	0.912
Specificity	0.550	0.703	0.802	0.728	0.902	0.916	0.875	0.905	0.893	0.890
F score	0.734	0.738	0.775	0.754	0.887	0.906	0.877	0.899	0.888	0.902
Youden	0.390	0.461	0.559	0.497	0.777	0.813	0.753	0.798	0.776	0.802
AUROC	0.782	0.817	0.846	0.749	0.931	0.966	0.889	0.964	0.951	0.964

B

Strength, Tract Count

	NB	Log	MP	SVM	kNN	Ada	J48	RF	Bag	KStar
Accuracy	0.730	0.724	0.797	0.763	0.863	0.914	0.851	0.928	0.901	0.888
Precision	0.697	0.724	0.806	0.751	0.855	0.913	0.854	0.937	0.896	0.866
Sensitivity	0.815	0.727	0.781	0.787	0.874	0.915	0.847	0.917	0.907	0.919
Specificity	0.644	0.722	0.812	0.738	0.852	0.913	0.855	0.938	0.895	0.857
F score	0.751	0.725	0.794	0.769	0.865	0.914	0.851	0.927	0.902	0.892
Youden	0.459	0.449	0.593	0.525	0.726	0.828	0.702	0.855	0.802	0.776
AUROC	0.805	0.807	0.858	0.763	0.920	0.966	0.869	0.975	0.956	0.959

Strength, Quantitative Anisotropy

	NB	Log	MP	SVM	kNN	Ada	J48	RF	Bag	KStar
Accuracy	0.725	0.719	0.792	0.743	0.818	0.916	0.821	0.929	0.861	0.897
Precision	0.696	0.725	0.792	0.732	0.815	0.920	0.824	0.933	0.864	0.905
Sensitivity	0.800	0.707	0.791	0.767	0.824	0.910	0.817	0.924	0.857	0.886
Specificity	0.650	0.732	0.792	0.718	0.812	0.921	0.825	0.934	0.865	0.907
F score	0.745	0.716	0.792	0.749	0.819	0.915	0.821	0.929	0.861	0.896
Youden	0.450	0.439	0.583	0.485	0.636	0.831	0.642	0.858	0.722	0.793
AUROC	0.801	0.789	0.808	0.763	0.820	0.966	0.843	0.979	0.906	0.962

C

Clustering Coefficient, Tract Count

	NB	Log	MP	SVM	kNN	Ada	J48	RF	Bag	KStar
Accuracy	0.609	0.775	0.805	0.766	0.852	0.901	0.863	0.920	0.889	0.893
Precision	0.569	0.770	0.799	0.768	0.846	0.899	0.863	0.917	0.889	0.867
Sensitivity	0.903	0.783	0.815	0.762	0.860	0.904	0.863	0.923	0.888	0.927
Specificity	0.315	0.766	0.795	0.769	0.843	0.898	0.863	0.916	0.889	0.858
F score	0.698	0.777	0.807	0.765	0.853	0.901	0.863	0.920	0.889	0.896
Youden	0.218	0.549	0.610	0.531	0.703	0.802	0.726	0.839	0.777	0.785
AUROC	0.772	0.851	0.866	0.766	0.905	0.957	0.885	0.975	0.953	0.958

Clustering Coefficient, Quantitative Anisotropy

	NB	Log	MP	SVM	kNN	Ada	J48	RF	Bag	KStar
Accuracy	0.696	0.747	0.796	0.757	0.831	0.921	0.885	0.912	0.891	0.901
Precision	0.653	0.738	0.800	0.750	0.825	0.920	0.878	0.915	0.895	0.890
Sensitivity	0.838	0.766	0.789	0.771	0.840	0.922	0.894	0.907	0.886	0.916
Specificity	0.553	0.727	0.802	0.742	0.821	0.920	0.875	0.916	0.896	0.886
F score	0.734	0.752	0.795	0.760	0.832	0.921	0.886	0.911	0.891	0.903
Youden	0.391	0.493	0.591	0.513	0.661	0.842	0.769	0.823	0.782	0.802
AUROC	0.780	0.828	0.858	0.757	0.883	0.974	0.905	0.969	0.950	0.964

D

Local Efficiency, Tract Count

	NB	Log	MP	SVM	kNN	Ada	J48	RF	Bag	KStar
Accuracy	0.712	0.746	0.805	0.763	0.863	0.922	0.885	0.911	0.897	0.899
Precision	0.691	0.744	0.799	0.763	0.855	0.920	0.872	0.920	0.904	0.885
Sensitivity	0.770	0.750	0.815	0.763	0.875	0.924	0.903	0.901	0.888	0.916
Specificity	0.654	0.742	0.795	0.763	0.851	0.920	0.867	0.921	0.906	0.881
F score	0.728	0.747	0.807	0.763	0.865	0.922	0.887	0.910	0.896	0.900
Youden	0.424	0.492	0.610	0.526	0.726	0.844	0.770	0.822	0.794	0.797
AUROC	0.787	0.827	0.866	0.763	0.921	0.966	0.897	0.964	0.952	0.965

Local Efficiency, Quantitative Anisotropy

	NB	Log	MP	SVM	kNN	Ada	J48	RF	Bag	KStar
Accuracy	0.664	0.743	0.818	0.759	0.877	0.884	0.871	0.906	0.879	0.914
Precision	0.623	0.728	0.817	0.770	0.879	0.887	0.870	0.910	0.886	0.903
Sensitivity	0.832	0.776	0.820	0.739	0.875	0.881	0.872	0.900	0.869	0.927
Specificity	0.496	0.710	0.816	0.779	0.879	0.887	0.869	0.911	0.888	0.900
F score	0.713	0.752	0.819	0.754	0.877	0.884	0.871	0.905	0.878	0.915
Youden	0.328	0.486	0.636	0.518	0.754	0.768	0.741	0.811	0.757	0.827
AUROC	0.785	0.817	0.875	0.759	0.922	0.943	0.879	0.964	0.949	0.972

Figure S2. Heatmap of the predictive performance indicators for training and validation of the ten machine learning models trained with the global graph measures as well as with the local graph measures degree (**A**; binary) and strength (**B**), clustering coefficient (**C**), and local efficiency (**D**) weighted with the parameter tract count and quantitative anisotropy, respectively. The color codes for accuracy, precision, sensitivity, specificity, and F-score are identical, ranging from minimum to maximum values. Separate color codes exist for Youden index, and AUROC due to their different value ranges.

A

Betweenness Centrality, Tract Count

	NB	Log	MP	SVM	kNN	Ada	J48	RF	Bag	KStar
Accuracy	0.663	0.786	0.813	0.785	0.880	0.898	0.827	0.918	0.878	0.933
Precision	0.620	0.778	0.805	0.797	0.886	0.905	0.835	0.932	0.881	0.930
Sensitivity	0.845	0.799	0.826	0.765	0.872	0.890	0.814	0.901	0.875	0.935
Specificity	0.481	0.772	0.800	0.805	0.887	0.906	0.839	0.934	0.881	0.930
F score	0.715	0.789	0.816	0.781	0.879	0.897	0.825	0.916	0.878	0.933
Youden	0.326	0.571	0.626	0.570	0.759	0.796	0.653	0.835	0.756	0.865
AUROC	0.792	0.856	0.880	0.785	0.925	0.955	0.847	0.970	0.941	0.981

Betweenness Centrality, Quantitative Anisotropy

	NB	Log	MP	SVM	kNN	Ada	J48	RF	Bag	KStar
Accuracy	0.707	0.766	0.833	0.781	0.892	0.909	0.852	0.920	0.864	0.943
Precision	0.671	0.759	0.842	0.768	0.897	0.912	0.857	0.927	0.875	0.937
Sensitivity	0.813	0.780	0.819	0.805	0.886	0.906	0.844	0.912	0.848	0.949
Specificity	0.600	0.752	0.846	0.756	0.898	0.912	0.859	0.928	0.879	0.936
F score	0.735	0.770	0.831	0.786	0.892	0.909	0.851	0.920	0.862	0.943
Youden	0.413	0.532	0.665	0.561	0.784	0.818	0.703	0.840	0.727	0.885
AUROC	0.782	0.832	0.882	0.781	0.935	0.968	0.867	0.971	0.940	0.986

B

Eigenvector Centrality, Tract Count

	NB	Log	MP	SVM	kNN	Ada	J48	RF	Bag	KStar
Accuracy	0.678	0.773	0.831	0.765	0.886	0.926	0.877	0.926	0.885	0.896
Precision	0.627	0.773	0.840	0.785	0.873	0.936	0.876	0.919	0.879	0.885
Sensitivity	0.883	0.772	0.817	0.732	0.902	0.914	0.877	0.935	0.892	0.911
Specificity	0.473	0.773	0.844	0.799	0.869	0.937	0.876	0.917	0.877	0.881
F score	0.733	0.773	0.829	0.757	0.888	0.925	0.877	0.927	0.886	0.898
Youden	0.356	0.545	0.661	0.531	0.771	0.851	0.753	0.852	0.769	0.792
AUROC	0.800	0.850	0.884	0.765	0.936	0.970	0.882	0.977	0.945	0.963

Eigenvector Centrality, Quantitative Anisotropy

	NB	Log	MP	SVM	kNN	Ada	J48	RF	Bag	KStar
Accuracy	0.736	0.777	0.819	0.766	0.861	0.909	0.875	0.920	0.893	0.896
Precision	0.738	0.776	0.822	0.753	0.858	0.906	0.879	0.924	0.907	0.897
Sensitivity	0.733	0.780	0.813	0.791	0.866	0.913	0.869	0.916	0.875	0.894
Specificity	0.740	0.774	0.824	0.740	0.856	0.905	0.880	0.924	0.910	0.897
F score	0.735	0.778	0.818	0.772	0.862	0.910	0.874	0.920	0.891	0.896
Youden	0.473	0.554	0.637	0.531	0.722	0.818	0.749	0.840	0.785	0.791
AUROC	0.797	0.854	0.888	0.766	0.921	0.967	0.887	0.972	0.950	0.961

C

PageRank Centrality, Tract Count

	NB	Log	MP	SVM	kNN	Ada	J48	RF	Bag	KStar
Accuracy	0.750	0.774	0.822	0.775	0.847	0.911	0.871	0.910	0.868	0.903
Precision	0.732	0.772	0.811	0.780	0.860	0.911	0.874	0.909	0.877	0.904
Sensitivity	0.791	0.779	0.840	0.767	0.828	0.911	0.866	0.910	0.855	0.901
Specificity	0.709	0.769	0.804	0.783	0.865	0.911	0.875	0.909	0.880	0.904
F score	0.760	0.776	0.825	0.774	0.844	0.911	0.870	0.910	0.866	0.903
Youden	0.500	0.548	0.644	0.550	0.693	0.822	0.741	0.819	0.735	0.805
AUROC	0.812	0.855	0.878	0.775	0.899	0.961	0.873	0.971	0.938	0.965

PageRank Centrality, Quantitative Anisotropy

	NB	Log	MP	SVM	kNN	Ada	J48	RF	Bag	KStar
Accuracy	0.741	0.751	0.809	0.770	0.833	0.907	0.851	0.926	0.869	0.890
Precision	0.737	0.747	0.821	0.766	0.834	0.917	0.858	0.933	0.880	0.884
Sensitivity	0.750	0.759	0.791	0.779	0.831	0.894	0.840	0.917	0.855	0.897
Specificity	0.732	0.742	0.827	0.761	0.834	0.919	0.861	0.934	0.883	0.882
F score	0.743	0.753	0.806	0.773	0.833	0.906	0.849	0.925	0.867	0.891
Youden	0.482	0.501	0.618	0.540	0.665	0.813	0.701	0.851	0.738	0.779
AUROC	0.811	0.822	0.874	0.770	0.896	0.967	0.860	0.977	0.936	0.958

D

Eccentricity, Tract Count

	NB	Log	MP	SVM	kNN	Ada	J48	RF	Bag	KStar
Accuracy	0.715	0.728	0.870	0.760	0.908	0.908	0.861	0.911	0.867	0.962
Precision	0.718	0.720	0.870	0.759	0.923	0.911	0.866	0.910	0.865	0.962
Sensitivity	0.708	0.748	0.870	0.762	0.889	0.903	0.854	0.911	0.870	0.961
Specificity	0.722	0.708	0.870	0.757	0.926	0.912	0.867	0.910	0.864	0.962
F score	0.713	0.733	0.870	0.761	0.906	0.907	0.860	0.911	0.868	0.962
Youden	0.430	0.456	0.740	0.519	0.815	0.815	0.721	0.821	0.734	0.923
AUROC	0.795	0.806	0.903	0.760	0.943	0.965	0.873	0.969	0.934	0.994

Eccentricity, Quantitative Anisotropy

	NB	Log	MP	SVM	kNN	Ada	J48	RF	Bag	KStar
Accuracy	0.723	0.753	0.857	0.762	0.877	0.920	0.841	0.920	0.887	0.949
Precision	0.709	0.754	0.861	0.754	0.886	0.925	0.850	0.933	0.884	0.948
Sensitivity	0.759	0.752	0.851	0.779	0.864	0.913	0.828	0.905	0.891	0.949
Specificity	0.687	0.754	0.862	0.745	0.889	0.926	0.853	0.935	0.883	0.948
F score	0.733	0.753	0.856	0.766	0.875	0.919	0.839	0.919	0.888	0.949
Youden	0.446	0.506	0.713	0.524	0.753	0.839	0.681	0.840	0.774	0.897
AUROC	0.795	0.813	0.897	0.762	0.924	0.974	0.857	0.973	0.949	0.988

Figure S3. Heatmap of the predictive performance indicators for training and validation of the ten machine learning models trained with the local graph measures betweenness centrality (**A**), eigenvector centrality (**B**), PageRank centrality (**C**), and eccentricity (**D**) weighted with the parameter tract count and quantitative anisotropy, respectively. The color codes for accuracy, precision, sensitivity, specificity, and F-score are identical, ranging from minimum to maximum values. Separate color codes exist for Youden index, and AUROC due to their different value ranges.

Table S3. Indicators of predictive performance for the top-performing models trained and validated using only demographic and clinical data.

	Accuracy	Precision	Sensitivity	Specificity	F-Score	Youden	AUROC
**Adaptive Boosting**	0.790	0.795	0.782	0.798	0.789	0.580	0.857
**Random Forest**	0.809	0.813	0.804	0.814	0.808	0.618	0.897
**KStar**	0.792	0.797	0.784	0.800	0.791	0.584	0.877

Table S4. Predictive performance indicators and 95% confidence intervals for the held-out internal testing using graph measures weighted with the parameter tract count.

Graph Measure	Model	Accuracy	Precision	Sensitivity	Specificity	Youden
Degree*	RF	0.862<br>[0.774, 0.919]	0.921<br>[0.792, 0.973]	0.795<br>[0.655, 0.888]	0.930<br>[0.814, 0.976]	0.725<br>[0.469, 0.864]
Strength	Ada	0.839<br>[0.748, 0.902]	0.813<br>[0.681, 0.898]	0.886<br>[0.760, 0.950]	0.791<br>[0.648, 0.886]	0.677<br>[0.408, 0.836]
Eigenvector Cent.	Ada	0.828<br>[0.735, 0.893]	0.822<br>[0.687, 0.907]	0.841<br>[0.706, 0.921]	0.814<br>[0.674, 0.903]	0.655<br>[0.380, 0.823]
Strength	RF	0.816<br>[0.722, 0.884]	0.850<br>[0.709, 0.929]	0.773<br>[0.630, 0.872]	0.860<br>[0.727, 0.934]	0.633<br>[0.357, 0.806]
Eccentricity	RF	0.805<br>[0.709, 0.874]	0.846<br>[0.703, 0.928]	0.750<br>[0.606, 0.854]	0.860<br>[0.727, 0.934]	0.610<br>[0.333, 0.789]
Between. Cent.	RF	0.805<br>[0.709, 0.874]	0.829<br>[0.687, 0.915]	0.773<br>[0.630, 0.872]	0.837<br>[0.700, 0.919]	0.610<br>[0.330, 0.790]
Eigenvector Cent.	RF	0.805<br>[0.709, 0.874]	0.829<br>[0.687, 0.915]	0.773<br>[0.630, 0.872]	0.837<br>[0.700, 0.919]	0.610<br>[0.330, 0.790]
Clustering Coeff.	RF	0.782<br>[0.684, 0.855]	0.766<br>[0.628, 0.864]	0.818<br>[0.680, 0.905]	0.744<br>[0.598, 0.851]	0.562<br>[0.278, 0.756]
Eccentricity	Ada	0.782<br>[0.684, 0.855]	0.838<br>[0.689, 0.923]	0.705<br>[0.558, 0.818]	0.860<br>[0.727, 0.934]	0.565<br>[0.285, 0.753]
Global*	RF	0.782<br>[0.684, 0.855]	0.879<br>[0.727, 0.952]	0.659<br>[0.511, 0.781]	0.970<br>[0.784, 0.963]	0.566<br>[0.295, 0.744]
Degree*	KStar	0.782<br>[0.684, 0.855]	0.791<br>[0.648, 0.886]	0.773<br>[0.630, 0.872]	0.791<br>[0.648, 0.886]	0.564<br>[0.278, 0.757]
Local Efficiency	Ada	0.770<br>[0.671, 0.846]	0.722<br>[0.591, 0.824]	0.886<br>[0.760, 0.950]	0.651<br>[0.502, 0.776]	0.537<br>[0.262, 0.726]
Clustering Coeff.	Ada	0.770<br>[0.671, 0.846]	0.773<br>[0.630, 0.872]	0.773<br>[0.630, 0.872]	0.767<br>[0.623, 0.868]	0.540<br>[0.253, 0.740]
Local Efficiency	RF	0.759<br>[0.659, 0.836]	0.767<br>[0.623, 0.868]	0.750<br>[0.606, 0.854]	0.767<br>[0.623, 0.868]	0.517<br>[0.228, 0.723]
PageRank Cent.	RF	0.724<br>[0.622, 0.807]	0.727<br>[0.582, 0.837]	0.727<br>[0.582, 0.837]	0.721<br>[0.573, 0.833]	0.448<br>[0.155, 0.669]
Degree*	Ada	0.724<br>[0.622, 0.807]	0.750<br>[0.598, 0.858]	0.682<br>[0.534, 0.800]	0.767<br>[0.623, 0.868]	0.449<br>[0.157, 0.669]
Clustering Coeff.	KStar	0.713<br>[0.610, 0.797]	0.656<br>[0.530, 0.763]	0.909<br>[0.788, 0.964]	0.512<br>[0.368, 0.654]	0.421<br>[0.156, 0.618]
Strength	KStar	0.713<br>[0.610, 0.797]	0.673<br>[0.541, 0.782]	0.841<br>[0.706, 0.921]	0.581<br>[0.433, 0.716]	0.422<br>[0.140, 0.637]
Local Efficiency	KStar	0.713<br>[0.610, 0.797]	0.694<br>[0.555, 0.805]	0.773<br>[0.630, 0.872]	0.651<br>[0.502, 0.776]	0.424<br>[0.132, 0.647]
PageRank Cent.	Ada	0.690<br>[0.586, 0.777]	0.730<br>[0.570, 0.846]	0.614<br>[0.466, 0.743]	0.767<br>[0.623, 0.868]	0.381<br>[0.089, 0.611]
Between. Cent.	KStar	0.690<br>[0.586, 0.777]	0.681<br>[0.538, 0.796]	0.727<br>[0.582, 0.837]	0.651<br>[0.502, 0.776]	0.378<br>[0.083, 0.612]
Between. Cent.	Ada	0.690<br>[0.586, 0.777]	0.698<br>[0.549, 0.814]	0.682<br>[0.534, 0.800]	0.698<br>[0.549, 0.814]	0.380<br>[0.083, 0.614]
Global*	Ada	0.678<br>[0.574, 0.767]	0.667<br>[0.525, 0.783]	0.727<br>[0.582, 0.837]	0.628<br>[0.479, 0.756]	0.355<br>[0.060, 0.593]
Global*	KStar	0.667<br>[0.562, 0.757]	0.683<br>[0.530, 0.804]	0.636<br>[0.489, 0.762]	0.698<br>[0.549, 0.814]	0.334<br>[0.038, 0.576]
Eigenvector Cent.	KStar	0.644<br>[0.539, 0.736]	0.623<br>[0.488, 0.741]	0.750<br>[0.606, 0.854]	0.535<br>[0.389, 0.675]	0.285<br>[-0.005,0.529]

Eccentricity	KStar	0.609<br>[0.504, 0.705]	0.596<br>[0.461, 0.718]	0.705<br>[0.558, 0.818]	0.512<br>[0.368, 0.654]	0.217<br>[-0.075,0.472]
PageRank Cent.	KStar	0.563<br>[0.459, 0.663]	0.558<br>[0.423, 0.684]	0.659<br>[0.511, 0.781]	0.465<br>[0.325, 0.611]	0.124<br>[-0.164,0.392]

* Global and degree measures are unweighted and identical for tract count and QA, yielding the same performance during held-out internal testing.

Table S5. Predictive performance indicators and 95% confidence intervals for the held-out internal testing using graph measures weighted with the parameter QA.

Graph Measure	Model	Accuracy	Precision	Sensitivity	Specificity	Youden
Strength	RF	0.874<br>[0.788, 0.928]	0.902<br>[0.775, 0.961]	0.841<br>[0.706, 0.921]	0.907<br>[0.784, 0.963]	0.748<br>[0.490, 0.884]
Clustering Coeff.	KStar	0.862<br>[0.774, 0.919]	0.820<br>[0.692, 0.902]	0.932<br>[0.818, 0.977]	0.791<br>[0.648, 0.886]	0.723<br>[0.466, 0.862]
Local Efficiency	RF	0.851<br>[0.761, 0.911	0.844<br>[0.712, 0.923]	0.864<br>[0.733, 0.936]	0.837<br>[0.700, 0.919]	0.701<br>[0.433, 0.855]
Clustering Coeff.	RF	0.839<br>[0.748, 0.902]	0.841<br>[0.706, 0.921]	0.841<br>[0.706, 0.921]	0.837<br>[0.700, 0.919]	0.678<br>[0.407, 0.840]
Clustering Coeff.	Ada	0.805<br>[0.709, 0.874]	0.800<br>[0.662, 0.891]	0.818<br>[0.680, 0.905]	0.791<br>[0.648, 0.886]	0.609<br>[0.328, 0.791]
PageRank Cent.	RF	0.805<br>[0.709, 0.874]	0.800<br>[0.662, 0.891]	0.818<br>[0.680, 0.905]	0.791<br>[0.648, 0.886]	0.609<br>[0.328, 0.791]
Eigenvector Cent.	Ada	0.793<br>[0.709, 0.874]	0.810<br>[0.667, 0.900]	0.773<br>[0.630, 0.872]	0.814<br>[0.674, 0.903]	0.587<br>[0.304, 0.774]
Local Efficiency	Ada	0.793<br>[0.709, 0.874]	0.825<br>[0.680, 0.913]	0.750<br>[0.606, 0.854]	0.837<br>[0.700, 0.919]	0.587<br>[0.304, 0.774]
PageRank Cent.	Ada	0.793<br>[0.709, 0.874]	0.825<br>[0.680, 0.913]	0.750<br>[0.606, 0.854]	0.837<br>[0.700, 0.919	0.587<br>[0.304, 0.774]
Strength	Ada	0.770<br>[0.671, 0.846]	0.750<br>[0.612, 0.851]	0.818<br>[0.680, 0.905]	0.721<br>[0.573, 0.833]	0.539<br>[0.253, 0.737]
Between. Cent.	RF	0.759<br>[0.659, 0.836]	0.725<br>[0.591, 0.829]	0.841<br>[0.706, 0.921]	0.674<br>[0.525, 0.795]	0.515<br>[0.231, 0.716]
Eigenvector Cent.	KStar	0.759<br>[0.659, 0.836]	0.745<br>[0.605, 0.847]	0.795<br>[0.655, 0.888]	0.721<br>[0.573, 0.833]	0.516<br>[0.228, 0.721]
Strength	KStar	0.759<br>[0.659, 0.836]	0.780<br>[0.633, 0.880]	0.727<br>[0.582, 0.837]	0.791<br>[0.648, 0.886]	0.518<br>[0.229, 0.722]
Between. Cent.	KStar	0.747<br>[0.647, 0.827]	0.720<br>[0.583, 0.825]	0.818<br>[0.680, 0.905]	0.674<br>[0.525, 0.795]	0.492<br>[0.206, 0.700]
Eigenvector Cent.	RF	0.736<br>[0.634, 0.817]	0.723<br>[0.582, 0.831]	0.773<br>[0.630, 0.872]	0.698<br>[0.549, 0.814]	0.471<br>[0.179, 0.686]
Between. Cent.	Ada	0.713<br>[0.610, 0.797]	0.721<br>[0.573, 0.833]	0.705<br>[0.558, 0.818]	0.721<br>[0.573, 0.833]	0.426<br>[0.131, 0.651]
Eccentricity	RF	0.701<br>[0.598, 0.787]	0.705<br>[0.558, 0.818]	0.705<br>[0.558, 0.818]	0.698<br>[0.549, 0.814]	0.403<br>[0.107, 0.632]
Eccentricity	KStar	0.701<br>[0.598, 0.787	0.714<br>[0.564, 0.828]	0.682<br>[0.534, 0.800]	0.721<br>[0.573, 0.833]	0.403<br>[0.108, 0.633]
Eccentricity	Ada	0.667<br>[0.562, 0.757]	0.674<br>[0.525, 0.795]	0.659<br>[0.511, 0.781]	0.674<br>[0.525, 0.795]	0.333<br>[0.037, 0.576]
Local Efficiency	KStar	0.644<br>[0.539, 0.736]	0.607<br>[0.481, 0.719]	0.841<br>[0.706, 0.921]	0.442<br>[0.304, 0.589]	0.283<br>[0.011, 0.510]
PageRank Cent.	KStar	0.621<br>[0.516, 0.715]	0.600<br>[0.468, 0.719]	0.750<br>[0.606, 0.854]	0.488<br>[0.346, 0.632]	0.238<br>[-0.048,0.487]

* Global and degree measures are unweighted and identical for tract count and QA, yielding the same performance during held-out internal testing.

Table S6. Indicators of predictive performance for the top-performing models tested using only demographic and clinical data.

	Accuracy	Precision	Sensitivity	Specificity	F-Score	Youden	AUROC
**Adaptive Boosting**	0.770	0.761	0.795	0.744	0.778	0.539	0.844
**Random Forest**	0.759	0.735	0.818	0.698	0.774	0.516	0.867
**KStar**	0.770	0.773	0.773	0.767	0.773	0.540	0.842

References

1. Sporns, O.; Kötter, R. Motifs in Brain Networks. *PLoS Biol.* **2004**, *2*, e369, doi:10.1371/journal.pbio.0020369.
